# A Step Forward in Prehospital Electrocardiogram Development∗

**DOI:** 10.1016/j.jacadv.2023.100441

**Published:** 2023-07-28

**Authors:** Mariam Jabara, Abhinav Sharma

**Affiliations:** a Centre for Outcomes Research and Evaluation, Research Institute of the McGill University Health Centre, Montreal, Quebec, Canada; b DREAM-CV Lab, McGill University Health Centre, McGill University, Montreal, Quebec, Canada; c Division of Cardiology, McGill University Health Centre, McGill University, Montreal, Quebec, Canada

**Keywords:** ECG, electrocardiogram, ischemia, prehospital ECG, portable ECG

**Figure:**
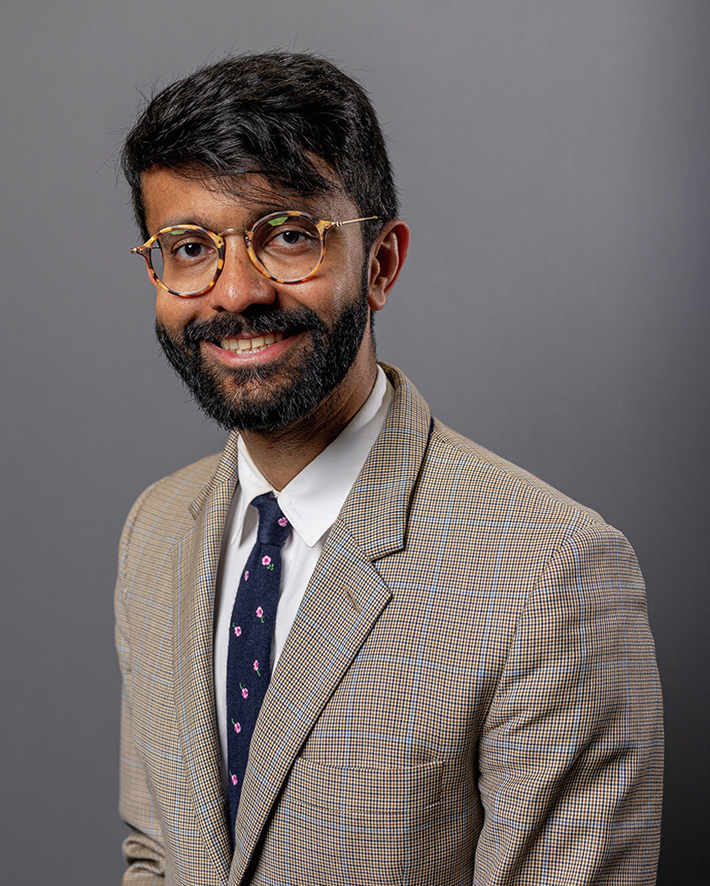

Cardiovascular diseases (CVDs) are the leading cause of death globally, and over three-quarters of CVD deaths occur in low- to middle-income countries.^1^ The timely management of the acute manifestations of CVD, such as myocardial ischemia, requires rapid assessment through electrocardiograms (ECGs). In the context of acute coronary syndrome, the acquisition of prehospital ECGs has been associated with greater use of reperfusion therapies, quicker reperfusion times, as well as significantly lower 30-day mortality risk.^2^^,^^3^ Ideally, the goal is to minimize the *door-to-balloon time*, which denotes the time from which the patient arrives at the emergency room until percutaneous coronary intervention is performed, where a catheter is used to inflate a small balloon in the occluded artery, restoring blood flow to the tissue. In practice, prehospital ECGs are an important tool in early detection of ischemia and in turn, can lead to improved door to balloon times. Many of the current tools for portable, home-based prehospital ECGs have been validated in the context of rhythmic disorders^4^; however, the validation of similar tools for acute events such as ischemia has yet to be investigated. Given the benefits of acquiring prehospital ECGs (either in professional settings or in the patient’s home) in acute coronary syndrome, a need for portable devices with adequate diagnostic accuracy of cardiac ischemia is presented. Furthermore, the disproportionate implications of CVD on low- to middle-income countries further solidifies the need to develop such portable devices as a potential avenue to increase access to care, improve patient outcomes, and promote timely detection of acute cardiovascular events in this population. Increased portability of devices remains an important milestone for increasing access to care in remote settings, where transportation and setup of devices such as the standard 12-lead ECG may be difficult.

## The miniECG: a new approach to prehospital ECGs for ischemia

In this issue of *JACC: Advances*, Vries et al^5^ propose the novel miniECG—a smartphone sized, multi-lead digital ECG device operable by a smartphone, and investigate the ability of this device to detect acute ischemia-induced ST-segment changes in the ECG. The authors conducted a proof-of-concept experiment in a porcine model by inducing myocardial infarction in eight animals and simultaneously taking ECG readings using the miniECG device and a standard 12-lead ECG before, during, and after the infarction. This study cites promising results with the miniECG device being positive for ischemia (ST-segment deviation ≥1 mm) for 99.7% of the occlusion time, while the 12-lead ECG was positive only 79.8% of the time. In addition to its portability, the device uses dry electrodes compared to standard disposable gel electrodes or patches of a 12-lead ECG, increasing sustainability and ease-of-use in nonclinical settings. Overall, this work addresses an important gap in prehospital care by proposing a portable, multi-lead ECG device that can detect ST-segment changes in ischemic events. The authors should be commended for the development of the proposed tool, evaluation, and clear and concise communication of their results in this study.

The authors found that there was a difference between the maximum ST-segment deviation between devices (with a higher amplitude in the miniECG); however, both devices show similar trends and patterns of peaks during occlusions and reperfusion. The study reports that the miniECG device was positive for ischemia 19.9% longer during occlusion than the gold standard. In all animals, the miniECG was positive for ischemia before the 12-lead ECG.

To contextualize the results, readers should be aware of the differences in anatomy of the porcine model vs human model, namely in the difference of the position of the heart within the chest and the electrophysiological differences. In this study, the placement of the 12-lead electrodes was slightly modified to create space in the central chest for the defibrillation paddles should the subjects require treatment for intolerable arrhythmias. Electrode placement has a strong effect on ST-segment deviations, and the placement of the electrodes further from the porcine heart in combination with the different anatomical position of the heart may explain the observed differences in ST-segment deviation magnitude. This does, however, render the results in this work challenging to compare to a 12-lead ECG in a human subject with standard placements. Additionally, the sinus node is highly innervated in the porcine model (compared to sparse innervation in humans), making the porcine heart more excitable, which may result in abnormal ECG readings relative to those acquired in clinical settings. Finally, the guidelines around what level of ST-segment elevation is pathological differs by age and sex in humans, therefore the authors may consider evaluating thresholds for ST-segment deviation other than 1 mm (eg, 0.5 mm, 2 mm) in future work.

## Future directions

**Figure 1:**
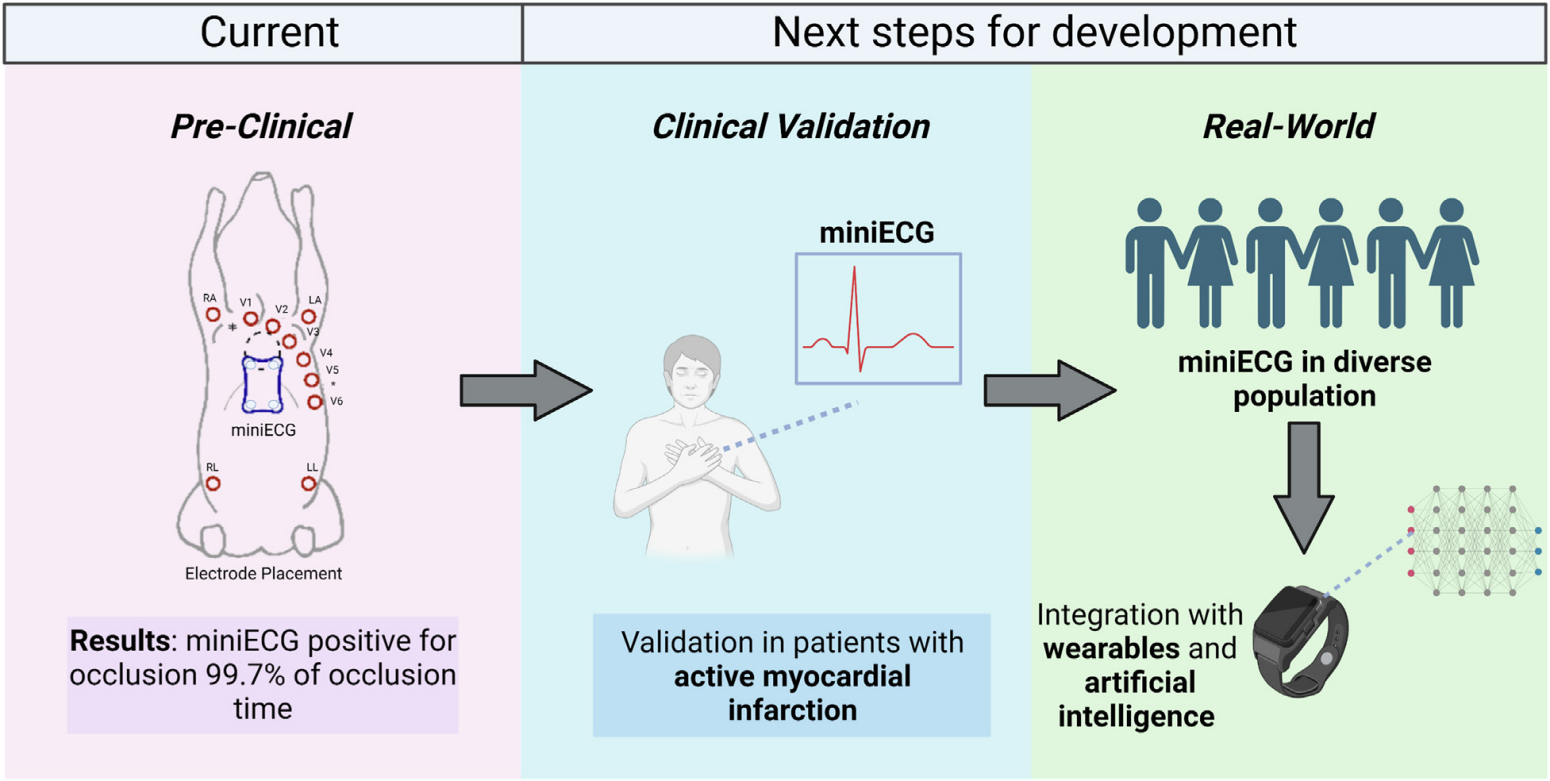
Next Steps for Development and Implementation of Prehospital ECG Description of the results found in the work of Vries et al^5^ on the miniECG, and next steps for clinical validation in patients with active myocardial infarction, real-world validation in a diverse population, and integration with wearable devices and artificial intelligence (AI) to improve patient outcomes.

Overall, the authors have proposed a novel tool with important initial data for a potential digital device that could provide a prehospital ECG for acute myocardial ischemia. Given that it is handheld, uses dry electrodes instead of disposable or gel patches, and is an easily portable device, it has the potential for use across ambulatory, patient, or remote field health care settings. The next steps would require validation of the device in an adult population before exploring the utility in a clinical setting. As seen in different CVDs such as heart failure, the use of digital devices may not improve data capture or provide more insights into the pathophysiologic conditions (Figure 1).^6^ Furthermore, the potential ease of use of such devices and a use-case wherein a symptomatic patient who is say, suffering chest pain, can use the device and be triaged appropriately represents a novel strategy for acute care management. Alternatively, the results can be sent to a physician for further evaluation. In future extensions of this work, the differences between the use of the tool by trained professionals versus patients will be interesting to uncover. The question is also raised on how this tool may be used for continuous remote patient monitoring (ie, not exclusively when the patient is symptomatic) to collect and interpret real-world ECG data.

The growing body of literature around portable ECG devices should motivate further investigation and hopefully increase the presence of similar devices across diverse settings. The use of artificial intelligence-based analysis of ECGs can enable the development of personalized home-based recommendations for the management of multiple acute and chronic diseases.^7^ Potentially integrating such devices with smartwatches that can collect ECG data may represent another novel strategy for home-based acute coronary syndrome detection. While patient physiological data are often collected in standardized clinical settings, the development and validation of such devices that collect data in ‘real-world’ settings will also be critical to improve patient outcomes, particularly in the CVD population. This work is promising in both the context of acute manifestations of CVD and in the evaluation of its chronic effects in nonclinical settings.

## Funding support and author disclosures

Ms Jabara is supported by a 10.13039/501100004489MITACS IT30282 award. Dr Sharma is supported by 10.13039/501100000024CIHR grant #451301 and a MEDTEQ^+^13-C CV Signature eSanté Grant; has received support from the Fonds de Recherche Santé Quebec (FRSQ) Junior 1 clinician scholars program, Alberta Innovates Health Solution, 10.13039/501100000860European Society of Cardiology young investigator grant, 10.13039/100016545Roche Diagnostics, Boehringer-Ingelheim, 10.13039/100004336Novartis, 10.13039/100004325AstraZeneca, Novo-Nordisk, 10.13039/100008497Boston Scientific, BMS-Pfizer, Akcea, and Takeda; and is an advisor for AREA19.
